# Supplementary material for: Acetyl-CoA synthetase (ACSS2) does not generate butyryl- and crotonyl-CoA
Source: Mol Metab. 2024 Feb 16;81:101903. doi: 10.1016/j.molmet.2024.101903 (PMC10906504; doi:10.1016/j.molmet.2024.101903)
Supplement: Multimedia component 1 [file mmc1.docx]

**Acetyl-CoA synthetase (ACSS2) does not generate butyryl- and crotonyl-CoA**

Nour Zeaiter^1^, Laura Belot^2^, Valérie Cunin^3^, Roland Abi Nahed^1^, Malgorzata Tokarska-Schlattner^1^, Audrey Le Gouellec^3^, Carlo Petosa^2^, Saadi Khochbin^4^*, Uwe Schlattner^1,5^*

**Supplemental material**

**Supplemental Figure 1: In vitro acetyl-CoA synthetase assay.** Preliminary experiment to check assay linearity and saturation, using yeast ACS2 and acetate as substrate. The reaction mix (100 μL) was quenched and ACS2 enzyme was precipitated by adding ice-cold 0.5 N perchloride acid (50 µL) and incubation on ice for 30 min. After centrifugation (3000 g, 12 min, 4°C), the supernatant containing acetyl-CoA product was neutralized with 5 M K_2_CO_3_ and centrifuged again (3000 g, 12 min, 4°C). Acetyl-CoA in the supernatant was quantified by HPLC [18].


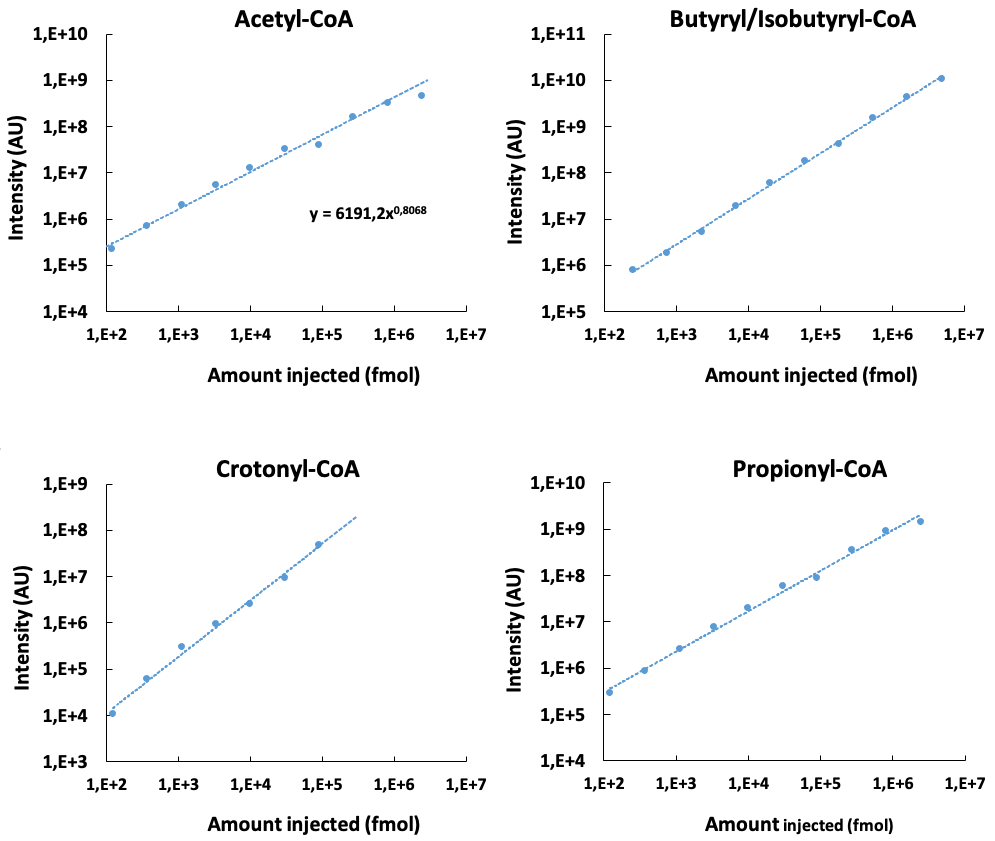


**Supplemental Figure 2: LC-MS/MS signal as a function of injected acyl-CoA standard.** A dilution series of a mixture of acyl-CoA standards was quantified by LC-MS/MS as published recently [18]. An exponential regression line is indicated. Note that data are presented with a double logarithmic scale.


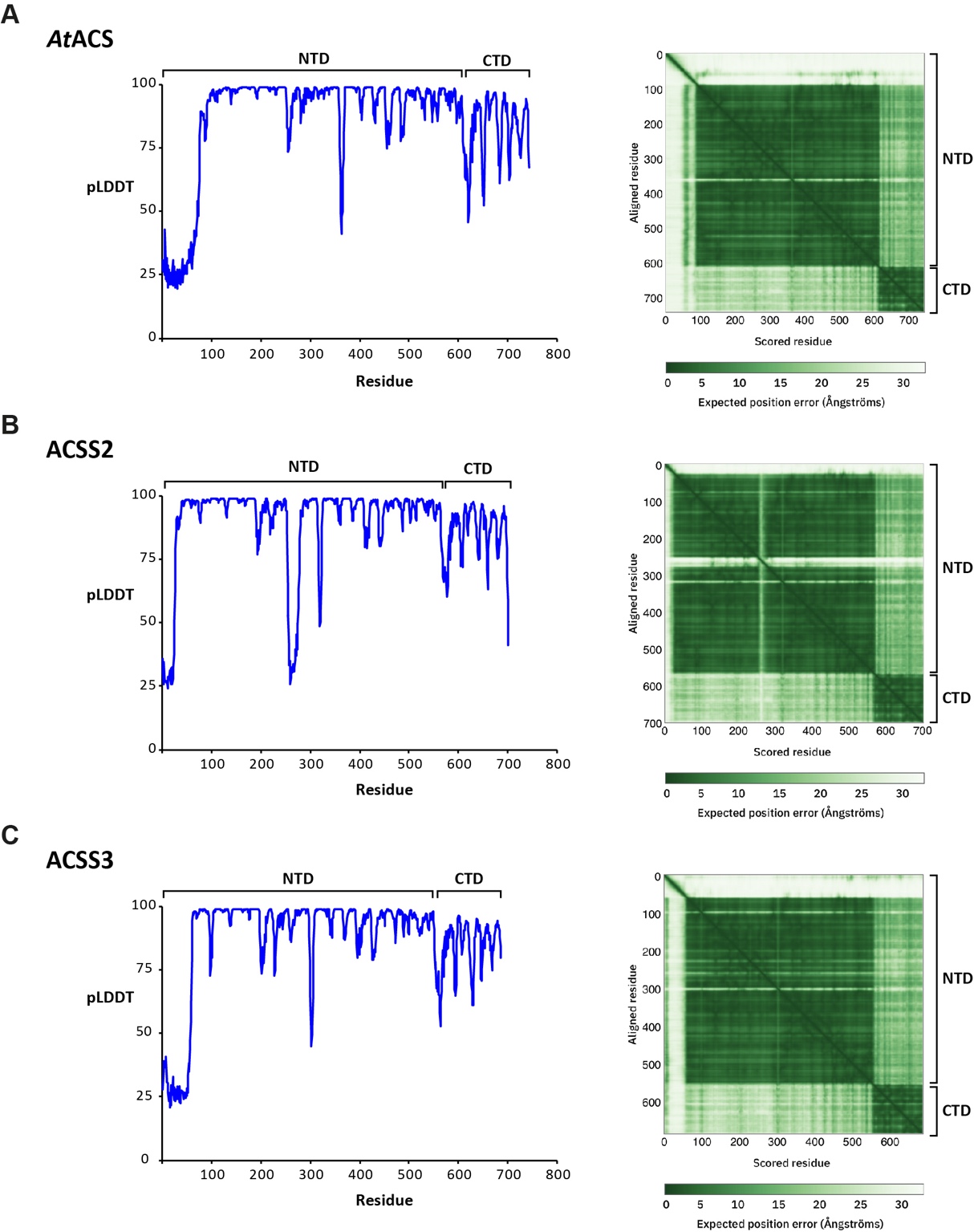


**Supplemental Figure 3. Confidence plots for AlphaFold models of ACS enzymes.** Plots of pLDDT score (left) and predicted aligned error (PAE) are shown for (A) *At*ACS, (B) human ACSS2 and (C) human ACSS3. The N-terminal domain (NTD) and C-terminal domain (CTD) limits are indicated.


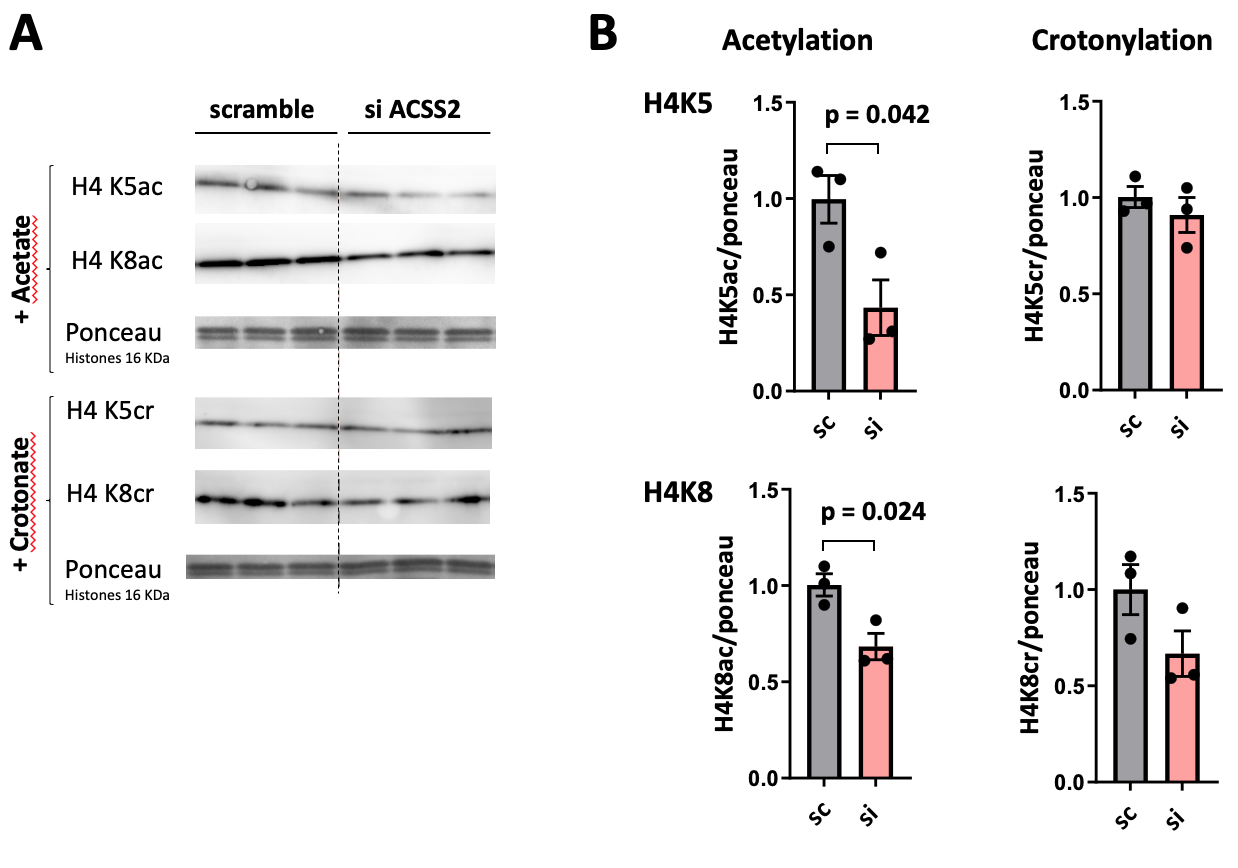


**Supplemental Figure 4: Effect of ACSS2 knock-down on the acylation status of histone 4 in presence of acetate or crotonate.** Cells were supplemented for 24 h with 5 mM acetate or 2,5 mM crotonate. (A) Immunoblot for acetylated or crotonylated H4K5 and H4K8; Ponceau stain of histones (at 16 kDa) is shown as loading control; full blots of loading controls are given in Suppl. Fig. 5. (B) Immunoblot quantification of (A) normalized to scramble. All data given as mean +/- SEM (*n*=3 independent HepG2 cultures). Sc, scramble siRNA; si-ACSS2, ACSS2-specific siRNA.


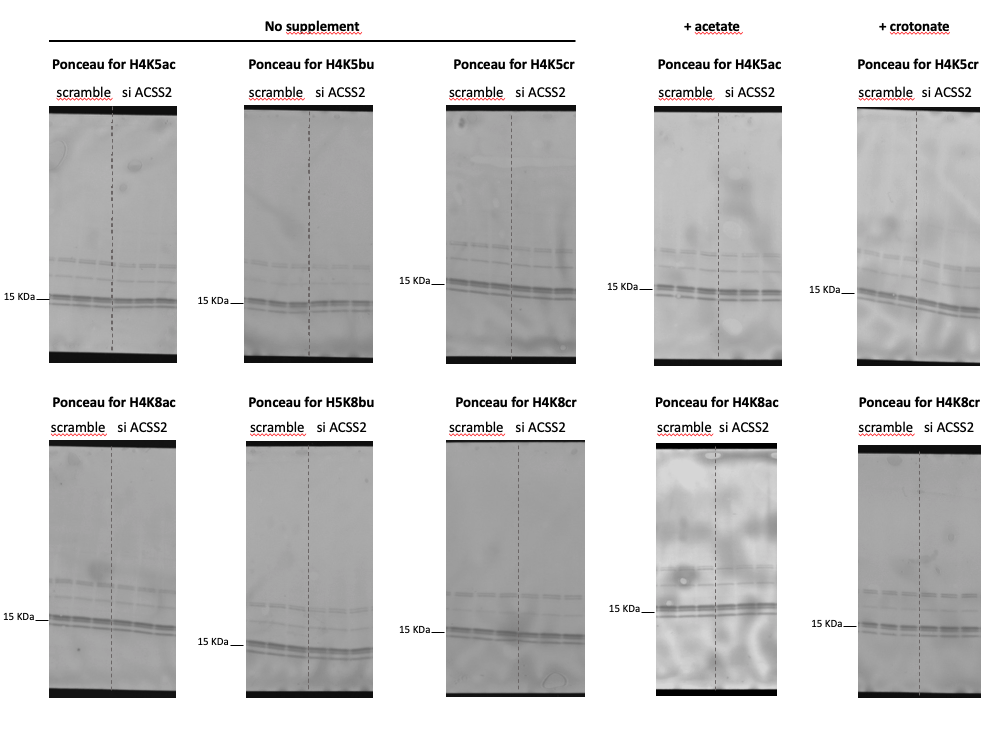


**Supplemental Figure 5. Immunoblot originals.** Ponceau stained blots used for quantification of loading controls (histone band at 16 kDa) in Fig. 1 and Suppl. Fig. 4. For abbreviations see these figures.


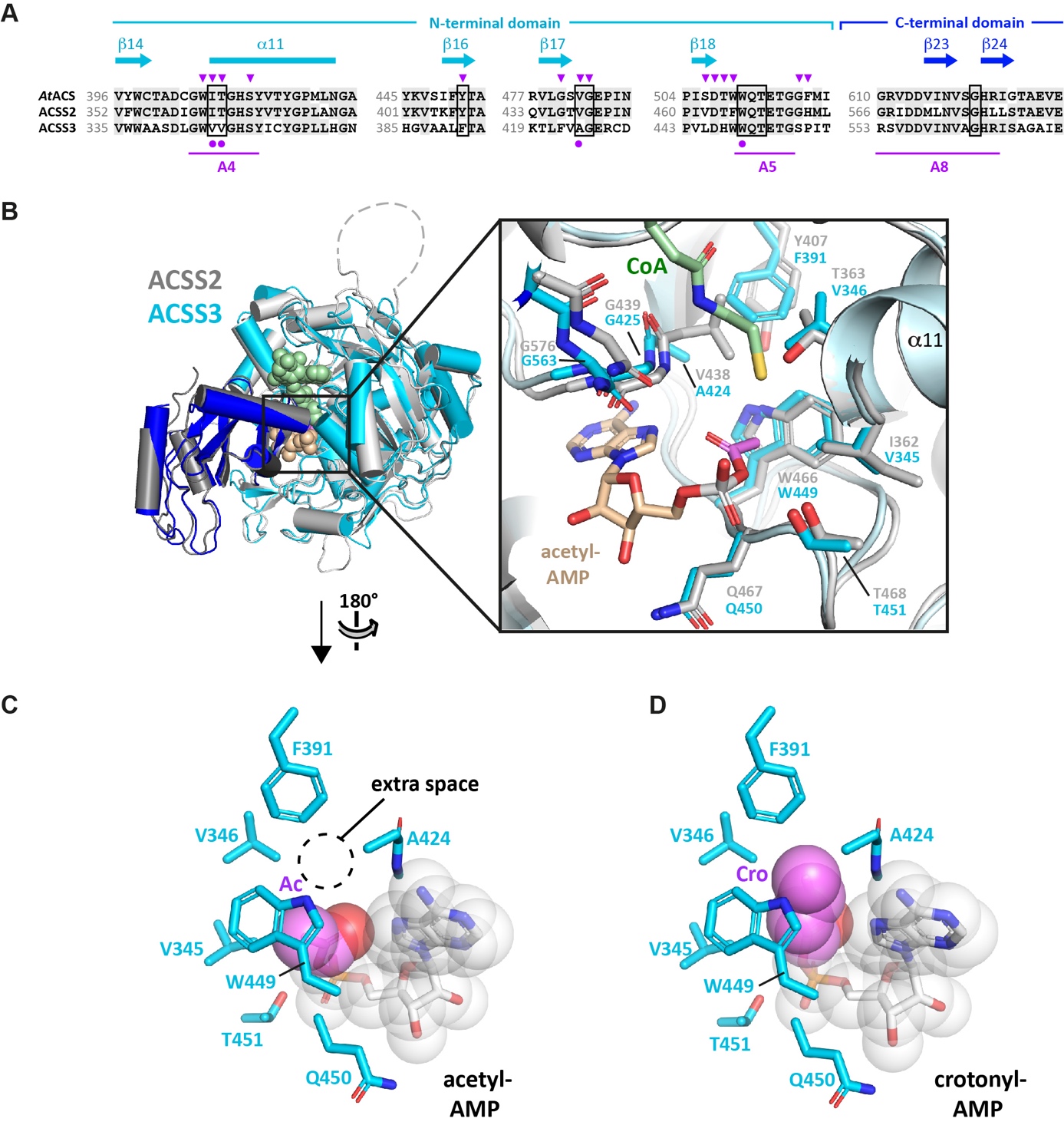


**Supplemental Figure 6. The predicted structure of the ACCS3 active site can feasibly accommodate butyryl- and crotonyl-AMP.** (**A**) Sequence alignment of *A. thaliana* ACS (*At*ACS) and human ACSS2 and ACSS3 residues spanning the active site. Secondary structure elements (cyan) and conserved motifs A4, A5 and A8 (magenta) of the AMP-forming family of acyl-CoA synthetases [32] are indicated. Residues implicated in substrate specificity [33] or proposed to form the carboxylate binding pocket [31] are indicated by arrowheads and circles, respectively. Boxed positions indicate residues predicted by structural modeling to lie within 6 Å of the acetyl group of acetyl-AMP. (**B**) Alignment of human ACSS2 and ACSS3 structural models. N- and C-terminal domains are shown in light and dark grey for ACSS2 and in cyan and blue for ACSS3, respectively. The structures were obtained by aligning the AlphaFold models for the N- and C-terminal domains onto the corresponding domains of *Salmonella enterica* ACS in the thioester-forming conformation (PDB 1PG4). *Inset*. Close-up view of the active site showing notable structural differences between ACSS2 and ACSS3. Residues boxed in (A) and the modeled CoA and acetyl-AMP ligands are indicated. (**C, D**) Predicted side chain environment of (C) an acetyl-AMP and (D) a crotonyl-AMP modeled in the active site of ACSS3. The acyl group carbon and oxygen atoms are in violete and red, respectively. The additional space next to the acetyl group is predicted to allow a bulkier butyryl or crotonyl group to fit comfortably within the active site.
